# Human footprint on estuarine tidal hydrodynamics

**DOI:** 10.1038/s41561-026-01969-4

**Published:** 2026-04-24

**Authors:** Joris G. W. Beemster, Stefan A. Talke, Dirk S. Van Maren, Nathalie Giloy, Anna Wünsche, Wei Zhang, Florent Grasso, Antonius J. F. Hoitink

**Affiliations:** 1 Department of Environmental Sciences, Wageningen University and Research, Wageningen, The Netherlands; 2 Department of Civil and Environmental Engineering, California Polytechnic State University, San Luis Obispo, San Luis Obispo, CA USA; 3 Ecosystems and Sediment Dynamics, Deltares, Delft, The Netherlands; 4 Faculty of Civil Engineering and Geosciences, Delft University of Technology, Delft, The Netherlands; 5 Shom, Brest, France; 6 Federal Waterways Engineering and Research Institute, Hamburg, Germany; 7 Coastal Geology and Sedimentology, Institute of Geosciences, Kiel University, Kiel, Germany; 8 State Key Laboratory of Hydrology-Water Resources and Hydraulic Engineering, Hohai University, Nanjing, China; 9 DYNECO/DHYSED, Ifremer, Plouzané, France

**Keywords:** Physical oceanography, Geomorphology

## Abstract

Natural estuarine morphology exerts strong control over tidal propagation. Human activities, such as dredging and land reclamation, modify the natural geometry, altering tidal dynamics and the ecosystems linked to them. Here we analyse changes in tidal dynamics, specifically the amplitude and propagation of tides, over decadal to centennial timescales, using archival maps, hydrographic surveys, tide gauge records and modern records from 25 estuaries worldwide, spanning the coast to their landward boundaries. Over the past two centuries, local interventions have typically amplified tidal ranges, accelerated tidal propagation and shifted tidal duration asymmetry. The most pronounced changes occurred far inland, often more than 100 km from the coast. Land reclamation and channel deepening are the most widespread and impactful interventions, affecting nearly all systems studied. The magnitude and inland location of maximum changes point to local human activities as the dominant drivers, exceeding the influence of long-term processes such as sea-level rise and natural subsidence and demonstrating that anthropogenic modifications have historically had the larger influence on estuarine water levels. Recognizing this human footprint opens opportunities for targeted local management strategies to reverse past changes, reduce flood risk and build resilience to climate change.

## Abstract (web summary)

Human activities such as dredging and land reclamation have pervasively altered the ways that tides propagate through large estuaries with negative ecological impacts, according to an analysis of recent and historical records.

## Main

Estuaries are dynamic environments at the interface between land and sea, shaped by the interaction of fluvial and tidal hydrodynamics, sediment transport and ecological processes^1^. Tidal motion exerts strong control over extreme water levels, sediment redistribution and salt intrusion and, in turn, affects estuarine morphodynamics^2^ and flood safety^3^. Human interventions increasingly disrupt tidal dynamics^3–5^, complicating estuarine management amid rising sea levels^6–9^ and changing weather patterns^10–13^.

Tidal propagation reflects how fast high-water peaks and the associated flood hazard travel landward. It is primarily governed by a balance between frictional energy losses at the bed and banks and the concentration of tidal energy caused by a landward reduction in cross-sectional area^14–16^. Tidal motion is damped and slowed down by friction but amplified and accelerated by cross-sectional convergence. Where the cross-sectional area reduces inland, waves amplify and propagate faster than the shallow-water wave speed, which increases with the root of depth. This often leads to flood dominance, where high-water crests advance faster than low-water troughs. In friction-dominated or weakly convergent estuaries, tidal damping dominates and tidal propagation is slower^14,17^. Storage of water in areas only inundated during high water reduces the strength of flood flows and can even lead to ebb dominance^2^.

During the past centuries, estuarine tides have been increasingly modified by a combination of natural and human-induced factors^18^. Human interventions such as dredging^19–22^, land reclamation^23–25^ and the construction of tidal barriers^26–28^ disrupt the balance between frictional damping and convergence, often favouring reduced attenuation by channel deepening and loss of surface area. Intended to facilitate navigation, flood protection or land use, these modifications have unintentionally modified estuarine tidal hydrodynamics and sediment dynamics^29,30^, with cascading effects on sediment transport^31^, salt intrusion^32^, water quality^33^, geomorphology^34,35^ and flood risk^36,37^.

Existing studies have investigated tidal changes at individual tide gauges^38–43^, within single estuaries^22,44–47^ or at a regional scale^4^. Comparative analyses spanning multiple estuaries that capture along-estuarine changes in tidal dynamics are lacking, despite previous studies illustrating how human interventions may profoundly influence the along-estuary distribution in tidal range. Global studies on tidal dynamics often focus on coastal stations^48,49^, overlooking the substantial changes that occur within estuaries. Inland stations, due to their relatively shallow depths, exhibit shorter tidal wavelength scales that approach the scale of human interventions, making them more sensitive to local changes through enhanced frictional effects and altered wave propagation^3,18^. Variations in geomorphology, tidal forcing and riverine influence, combined with limited long-term records that predate major interventions, have constrained our understanding of global estuarine tidal change^3^. A global comparative perspective can reveal which estuary types and zones are most sensitive to different kinds of human interventions and which changes are likely to enhance or compromise resilience.

**Fig. 1:**
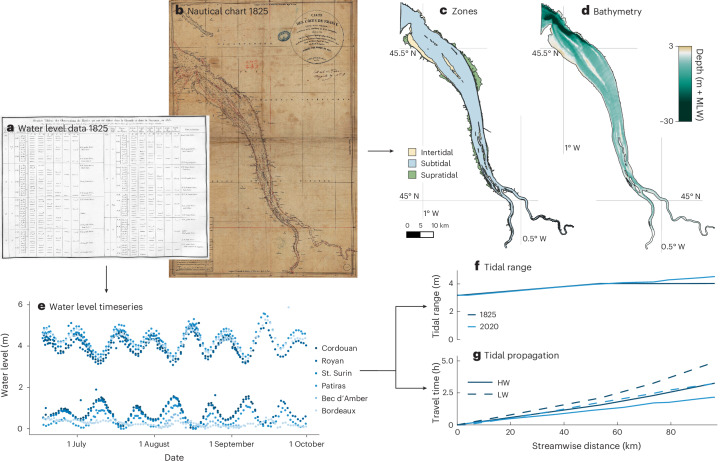
Example of the data collection and processing. **a**, Historical water level records for the Gironde^84^. **b**, Nautical chart of the Gironde^85^. **c**, Zonation of the Gironde Estuary (1825). **d**, Bathymetric map of the Gironde Estuary (1825). **e**, Extracted high–low water level time series for six stations along the Gironde Estuary. **f**, Along-channel tidal range profiles of the Gironde Estuary. **g**, Along-channel tidal propagation profiles of the Gironde Estuary. Solid lines indicate high-water propagation, whereas dashed lines represent low-water propagation. Credit: **a**,**b**, Service Hydrographique et Océanographique de la Marine (https://diffusion.shom.fr).

This article presents a global-scale comparative analysis of tidal hydrodynamics and human interventions using observations from 25 estuaries. We examine changes in water level and channel geometry since the nineteenth century and along the estuarine gradient, stretching from the mouth to the tidal limit. These estuaries are morphodynamically active, influenced by both tidal and riverine processes, and sufficiently long for pronounced tidal evolution along the estuary. Systems dominated by wave action, fjord-type estuaries and lagoonal environments are excluded because of their distinct hydrodynamics. A comprehensive perspective on changes in tides in human-influenced estuaries is obtained by analysing historical estuarine geometries, tidal ranges, high and low water levels, tidal propagation and tidal asymmetry (Methods). Our approach examines two periods: the earliest available records, providing a baseline of tidal dynamics and modern observations, which integrate the cumulative impact of major human interventions. Leveraging newly digitized historical datasets (for example, Fig. 1) and existing studies, we link observed changes to specific human activities, while acknowledging the potential influence of external drivers such as sea-level rise^50,51^, subsidence^52,53^, sediment supply^54,55^, estuarine morphodynamics^56^, altered river flow^57^ and extreme events^58,59^.

## Changes in tidal characteristics

**Fig. 2:**
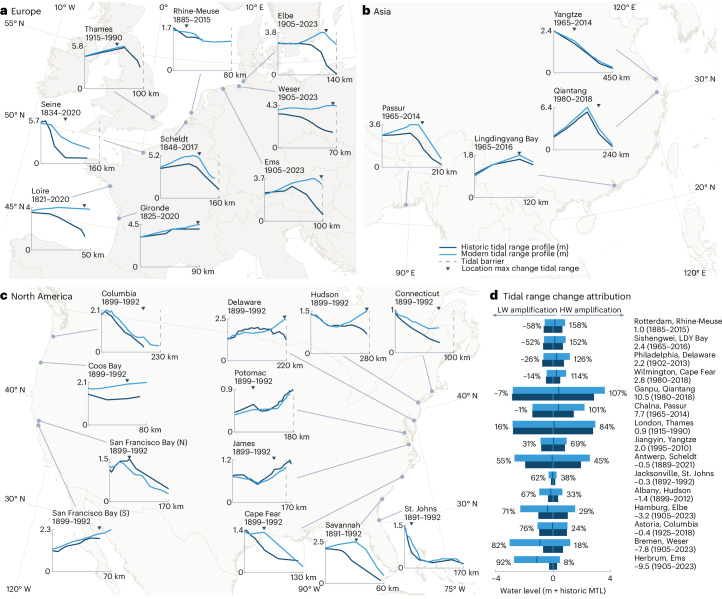
Tidal ranges in estuaries have amplified both by low-water decrease and high-water rise. **a**–**c**, Geographic distribution of the 25 estuaries included in this study across Europe (**a**), Asia (**b**) and North America (**c**). For each estuary, the historical (dark blue line) and modern (light blue line) tidal range profiles are plotted as a function of streamwise distance from the estuary mouth in kilometres. Where present, tidal barriers are indicated by vertical black dashed lines, typically located further inland rather than directly at the estuary mouth. The time periods for the historical and modern datasets are specified in the title of each subplot. N and S denote the northern and southern portions of San Francisco Bay, respectively, with the northern portion continuing into the Sacramento River. **d**, Attribution of tidal range changes to relative increases in mean high water (MHW) and decreases in mean low water (MLW) for selected tide gauges across the studied estuaries. The dark blue bars represent the historical tidal range, while the light blue bars indicate the modern tidal range. The percentages indicate the contribution of MHW or MLW changes to the total tidal range change, using the mean tide level (MTL) of the historical period as a reference. MTL is shown by vertical lines within the bars; its rate of change (mm yr^−1^) is shown below each tide gauge name. Tide gauges in **d** were selected for data quality, vertical datum consistency and inland location to isolate local human influences. The stations are intended as illustrative examples and are not necessarily representative of the entire estuary. Basemap data in **a**–**c** from the Global Self-consistent, Hierarchical, High-resolution Geography database (GSHHG; https://www.soest.hawaii.edu/pwessel/gshhg)^86^.

The most pronounced changes in the tidal range occur in upstream regions, particularly near tidal barriers and in zones previously characterized by strong attenuation of the tidal wave (Fig. 2). In 20 of 25 systems, the point of maximum absolute tidal range change lies landward of the historical maximum tidal range, reflecting the compounding effects of reduced friction and enhanced convergence as the tide moves upstream. The spatial pattern of tidal range amplification differs between estuaries: some exhibit gradual, along-channel increases (for example, the Scheldt and Loire), while others display more localized amplification (for example, the Delaware and Hudson). Near tidal barriers, indicated by dashed vertical lines in Fig. 2, reflection dynamics contribute to strong, localized tidal amplification, with increases exceeding 0.5 m in the Elbe, Weser, Ems, Hudson and Delaware. Regions with steep gradients in the tidal range, often due to frictional dampening, are especially sensitive to changes; even minor shifts in the along-channel profile can produce substantial local amplification, as observed in the Saint Johns Estuary^36^ and the Seine. Changes at estuary mouths are minor (max 0.28 m; median 0.04 m).

Tidal amplification is often driven by unequal shifts in high and low water (HW/LW): some estuaries are dominated by falling LW (for example, the Ems and Weser), others by rising HW (for example, the Thames and Passur; Fig. 2d). These variations can differ along the estuary. A drop in LW in the upper reaches can result from reduced river discharge^57,60,61^ or diminished frictional losses due to channel deepening^21,36^. In contrast, land reclamation disproportionately increases HW by reducing intertidal storage^62^. Asymmetric shifts in HW and LW alter the mean tide level (MTL, average of HW and LW), with implications for perceived sea-level trends. In our dataset, MTL change rates vary widely, from −9.5 to 10.5 mm yr^−1^ (Fig. 2d), exceeding local sea-level rise rates at the mouths of the estuaries (-1.3 to 2.0 mm yr^−1^; Extended Data Table 1; ref. ^63^) in several cases. This suggests that hydrodynamic adjustments to local interventions can dominate observed trends, particularly in upstream estuarine zones. Fully disentangling the effects of changing hydrodynamics, tidal asymmetry, effects of subsidence and data uncertainties on estuarine sea-level trends requires site-specific analyses^21,44^.

**Fig. 3:**
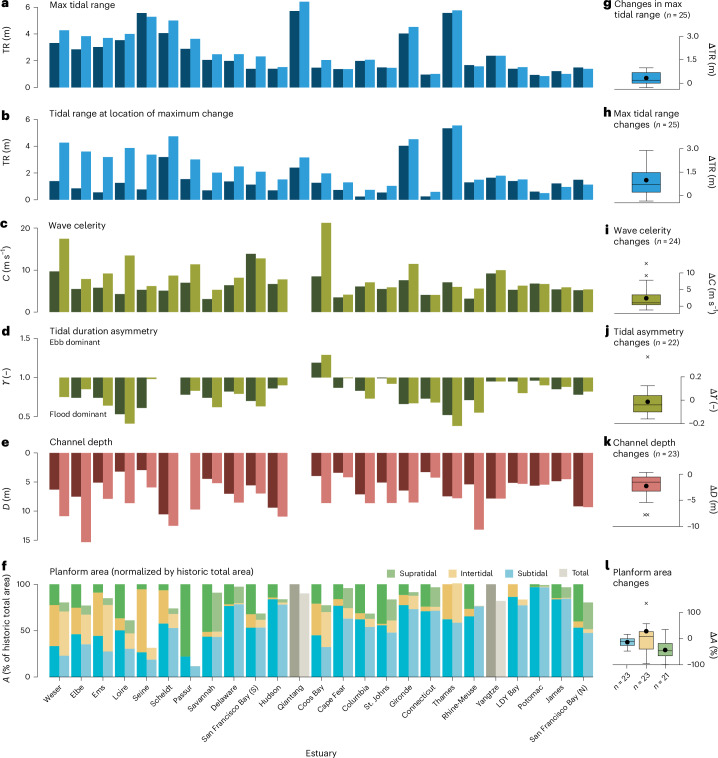
Key variables for the historic and modern conditions across the 25 estuaries. **a**–**f**, The variables include: maximum tidal range (**a**), tidal range at the location of maximum (absolute) change (**b**), wave celerity (the speed at which the tidal wave propagates through the estuary) (**c**), tidal duration asymmetry (**d**), channel depth (**e**) and planform area (normalized to the historic total area, separated into supratidal, intertidal and subtidal area) (**f**). Darker bars refer to the historic situation and lighter bars to modern conditions. **g**–**l**, Box-and-whisker plots show the distribution of differences (modern minus historic); boxes indicate the interquartile range (IQR) with the median, whiskers extend to ±1.5× IQR, small crosses denote outliers and black circles indicate mean changes. Sample sizes (*n*) are indicated in the panel subtitles (and below the boxplots in **l**). The estuaries are sorted based on changes in tidal range at the point of maximum absolute change, essentially ranking them from most to least tidal amplification. Grey shading in panel **f** indicates cases where only total area data were available.

**Extended Data Fig. 1:**
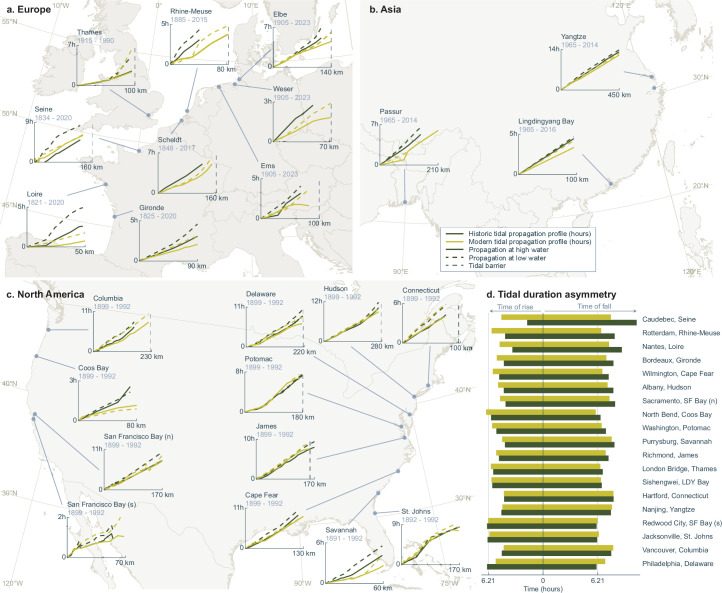
Tidal propagation in estuaries. (**a**-**c**) Geographic distribution of the estuaries analysed in this study across (**a**) Europe, (**b**) Asia, and (**c**) North America. For each estuary, the historical (dark green) and modern (light green) tidal propagation profiles are plotted as a function of streamwise distance from the estuary mouth (km). Solid lines represent high-water propagation, while dashed lines indicate low-water propagation. Estuaries where high water propagates faster than low water are considered flood dominant. Vertical black dashed lines mark the location of tidal barriers where present. The time periods for the historical and modern datasets are indicated in each subplot title. (**d**) Tidal duration asymmetry at selected stations, ranked from increasing flood dominance (bottom) to decreasing flood dominance (top). The bars on the left of the vertical zero line represent the duration of the rising tide, while those on the right indicate the duration of the falling tide. Stations where the falling tide lasts longer than the rising tide are classified as flood dominant. Historical tidal asymmetry is shown in dark green and modern asymmetry is shown in light green. The total duration of each bar represents a complete tidal cycle of 12 hours and 25 minutes. Tidal asymmetry plays a crucial role in sediment transport and consequently in estuarine morphological evolution. Basemap data in **a**–**c** from the GSHHG (https://www.soest.hawaii.edu/pwessel/gshhg)^86^.

Tidal propagation has accelerated markedly in most estuaries, with 22 of 24 systems showing an average increase of 2.03 m s^−1^ (38%; *σ* = ±3.33 m s^−1^; Fig. 3c), corresponding to a wavelength extension of ±90 km (Extended Data Fig. 1). Faster wave propagation also influences reflection dynamics, particularly in low-friction, low-convergence settings, where reflected waves attenuate less and amplify more effectively^21,28,31^. Changes in wave speed vary considerably between systems and are non-uniform along the estuary. Upstream reaches show the largest acceleration, sometimes exhibiting standing-wave behaviour (for example, the Ems and Coos Bay).

As the tidal wave propagates through the estuary, it deforms and becomes asymmetric. Except for Coos Bay, all estuaries analysed exhibit faster propagation of HW relative to LW, which we define here as flood dominance (Fig. 3d). Flood dominance increased in 11 of 22 systems, remained unchanged in two and decreased in nine, with Coos Bay becoming more ebb dominant (three estuaries lacked asymmetry data). The substantial variability in changes in tidal asymmetry probably reflects differences in dominant drivers. Reductions in the intertidal area consistently enhance flood dominance, whereas decreased river discharge tends to reduce it. Contrastingly, the impact of channel deepening remains more uncertain^64^.

These results demonstrate that most considered estuaries exhibit a strong reduction in friction losses, leading to a relative dominance of convergence, tidal amplification and accelerated wave propagation speeds. Although this study examines only two snapshots in time, one representing the earliest available data, predating most extensive modifications and the other recent observations, the observed changes highlight the cumulative impacts of reduced friction across estuarine systems. Many estuaries are probably still adapting to these changes, suggesting that further tidal changes are expected. Estuaries often exhibit an initial rapid response to interventions, followed by a slower, exponentially decaying phase of adjustment^65^. Overlapping human interventions frequently complicate these dynamics, resulting in a complex, nonlinear tidal evolution over time^3^.

## Relationship with anthropogenic interventions

**Fig. 4:**
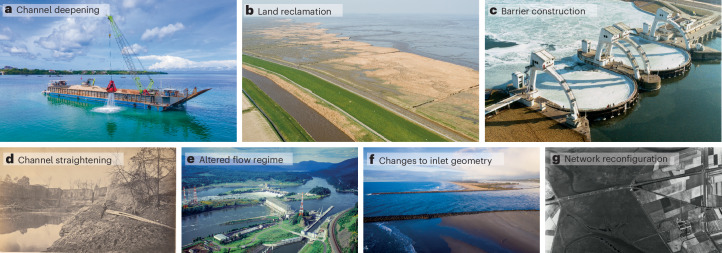
Contemporary human interventions in the estuaries studied. **a**, Channel deepening. Deepening of navigation channels through maintenance dredging to ensure port accessibility (Supplementary Fig. 4) and dredging for sand extraction has increased channel depths in many estuarine systems. **b**, Land reclamation in the Ems Estuary, Germany/the Netherlands. Historically, farmers and landowners stimulated sedimentation and wetland growth by constructing small brushwood dams, later embanking the areas to claim land. **c**, Barrier construction in the Rhine-Meuse estuary, the Netherlands. The Hagestein Weir and Lock Complex, shown here blocking upstream ice floes, marks the upstream limit of tidal propagation. **d**, Channel straightening in the James Estuary, USA. The construction of bend cut-offs, such as ‘The Dutch Gap’ during the American Civil War in 1864, shortened the estuary’s length by 17 km. **e**, Altered flow regime on the Columbia River, USA. The Bonneville Dam, along with other upstream dams, has reduced mean annual discharge and modified the annual hydrograph, lowering peak winter flows while increasing summer low flows. **f**, Changes to inlet geometry at Coos Bay, USA. The construction of jetties at the estuary mouth has increased the depth of the inlet and enhanced tidal penetration into the bay. **g**, Network reconfiguration in the Western Scheldt, the Netherlands. The Walcheren Causeway (Sloedam) disconnected the Western and Eastern Scheldt, eliminating upstream freshwater inflow to the Eastern Scheldt. Credit: **a**, tuzla/Shutterstock; **b**, Joop van Houdt, Rijkswaterstaat; **c**, Rijkswaterstaat; **d**, Andrew J. Russell, Library of Congress; **e**, Russ Heinl, Shutterstock; **f**, Manuela Durson, Shutterstock; **g**, Collection of the Zeeland Liberation Museum.

We identify seven key anthropogenic interventions influencing estuarine tidal dynamics in the estuaries studied (Fig. 4): channel deepening, land reclamation, tidal barrier construction, channel straightening, flow regime changes, inlet modifications and channel network reconfiguration. These interventions alter friction, convergence and storage, thereby impacting tidal attenuation and propagation.

**Extended Data Fig. 2:**
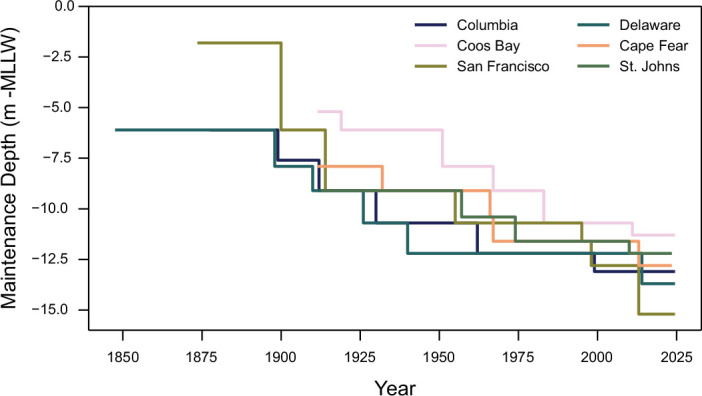
Stepwise increases in navigation channel maintenance depth for six U.S. estuaries. Maintenance depths are given relative to Mean Lower Low Water (MLLW). Historical depths were retrieved primarily from NOAA’s Office of Coast Survey^94^, supplemented with additional sources for the Columbia River Channel^67^, the Delaware River (mouth to Philadelphia)^47^, and the first two datapoints for San Francisco Bay (Oakland Harbor)^95^. Estuary coverage is defined as follows: Columbia (Columbia River Channel), Coos Bay (mouth to Isthmus Slough), San Francisco (Oakland Harbor), Delaware (mouth to Philadelphia), Cape Fear (mouth to Wilmington), and St. Johns (mouth to Jacksonville).

Channel deepening has led to substantial changes in the tidal dynamics of almost all estuaries analysed, primarily by reducing frictional damping, which amplifies the tidal range and accelerates wave propagation^4,16,36,47,64,66^. By improving discharge conveyance, deepened channels often reduce mean water levels upstream^21,36^. On average, mean channel depths increased from 6.0 m to 8.3 m below MTL, a 2.3 m (38%) rise, with a standard deviation of ±2.3 m, with several systems experiencing a more than twofold increase (Seine: 101%, Elbe: 104%, Coos Bay: 117%, Rhine-Meuse: 144% and Loire: 169%; Fig. 3k). These values represent estuary integrated depths; localized increases in navigational fairways are often much greater (Extended Data Fig. 2; refs. ^22,67^). These changes result primarily from dredging and subsequent morphological adaptation^65^.

Navigation depths have probably increased even more, as seen in the Columbia River Estuary, where the controlling depth rose from 6.1 m in 1878 to 13.1 m in 1999 (ref. ^67^). Systems with extensive deepening show the most pronounced increases in tidal range and propagation speed (Fig. 3), while those with minimal changes (for example, San Francisco North Bay, James and Potomac) exhibit weaker responses. Although (relative) sea-level rise can also alter channel depth by raising the water surface elevation, its contribution over the considered period appears limited. Estimates of sea-level rise near the estuary mouths (Extended Data Table 1) remain below 0.2 m, which is a minor contribution to the overall channel deepening.

Land reclamation has similarly altered tidal hydrodynamics by reducing estuarine storage, concentrating tidal energy within the remaining channel and amplifying high-water levels. On average, total estuarine planform area decreased by 21% ± 20% (Fig. 3l). The most substantial losses occurred in supratidal zones, defined here as areas situated above mean high water (MHW) that are still episodically flooded, such as during spring tides or storm surges, which declined by 49% ± 35%. Subtidal and intertidal areas decreased more modestly (14% ± 16% and 4% ± 50%, respectively). The latter includes high variability across estuaries and may reflect morphological adaptation or mapping uncertainty rather than a consistent global trend. One extreme outlier (St. Johns) showing 714% increase was excluded to avoid skewing the mean, probably reflecting mapping uncertainty and a small intertidal area. Changes to tidal datums strongly influence ecological zonation and can trigger major shifts in wetland type, particularly when such changes occur rapidly^68^. In many European estuaries, large-scale reclamation predates tide gauge records, suggesting its full impact on tidal dynamics is probably underestimated^23,34^.

Tidal barriers, defined here as inland structures or natural features that restrict tidal propagation, are present in 13 of 25 estuaries studied (Extended Data Table 2). Nine are anthropogenic (for example, storm surge barriers or weirs) and four are natural (for example, waterfalls). Natural barriers are found in the Delaware, James, Potomac and Connecticut, where geological features limit upstream tidal propagation. While full closures at the mouth would suppress tidal amplitudes entirely, inland barriers allow tides to persist seaward of the structure, often amplified due to reflection. Although typically localized, barrier impacts can extend system-wide depending on estuarine geometry, friction and proximity to resonance^28^. For example, in the Weser and Ems estuaries, barriers have contributed to marked tidal amplification. The degree of reflection is shaped by local geometry: in convergent estuaries, reflected energy attenuates rapidly, whereas in weakly convergent systems, reflected waves can travel further downstream.

River straightening and changes in the channel network or inlet geometry are less prevalent but contribute to localized changes in specific estuaries. Channel straightening was observed in six estuaries, with the James River experiencing the greatest reduction in estuarine length (17 km or 10%). Straightening primarily enhances tidal convergence by reducing channel length and flow resistance but is unlikely to strongly influence reflection dynamics^36^. Modifications to inlet geometry were identified in at least 11 estuaries and typically involve the construction of jetties or breakwaters to maintain navigable depths. By streamlining tidal flow and increasing inlet depth, these interventions reduce the ’choked’ nature of the inlet, allowing more tidal energy to penetrate the estuary^69^. Their hydrodynamic impact is most pronounced in systems with naturally restricted inlets. In the United States, inlet modifications, particularly jetty construction, have affected the majority of estuaries in our dataset, probably due to the prevalence of naturally restricted inlets. Notable alterations to channel networks were also observed. In the Scheldt and Savannah, connections between branches were closed. Other estuaries experienced an expansion of existing connections^25^ or construction of new connections. Estuaries such as the Ems and Loire have seen the loss or infilling of secondary channels due to land reclamation and deepening of primary channels^4,34^, transforming multichannel systems into predominantly single-channel configurations.

**Extended Data Fig. 3:**
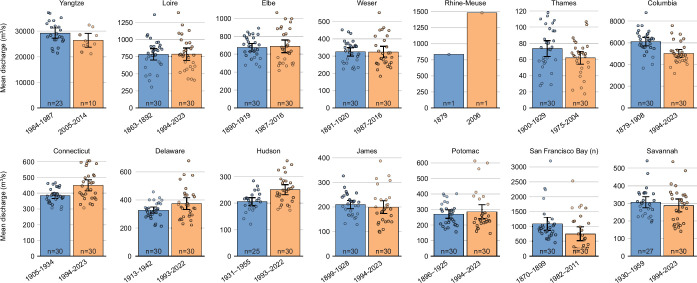
Changes in mean annual river discharge across estuaries. Each panel shows annual mean river discharge for two multi-decadal periods (earliest available data in blue, recent data in orange) across 14 estuaries with sufficient temporal coverage. Bars indicate historical and modern discharge estimates derived from long-term gauging records or reconstructed inflow series, with 95% confidence intervals where available. Semi-transparent dots show the individual annual mean discharge values within each period; dots are horizontally offset (jittered) for clarity to avoid overlap. The sample size is indicated at the bottom of the bar. Where possible, 30-year averaging periods were used to account for interannual variability. Independent vertical scales are applied to accommodate the wide range in river magnitudes.

Our analysis of mean annual discharge reveals substantial variability across estuaries (Extended Data Fig. 3). The Rhine-Meuse system shows the largest increase (+78% (ref. ^70^)), while significant decreases occurred in the Sacramento (−31% ± 30%, 95% CI) and Columbia (−18% ± 9%, 95% CI) rivers, based on comparisons between the late nineteenth and early twenty-first centuries. These results align with previous estimates^44,71,72^. Discharge generally has the greatest effects in the upstream reaches of estuaries, where riverine and tidal flows are of comparable magnitude. Large changes in discharge may have a limited impact on tidal dynamics when tidal velocities are much larger than river discharge^71^. High discharges typically suppress tidal amplitudes upstream by increasing bottom friction, but this relationship can be highly nonlinear^57,61,73^. Elevated river discharge slows the propagation of tidal waves by reducing their celerity^17^. No consistent trend in annual discharge emerged across the systems studied, which is why the global trend of tidal amplification cannot be attributed to river discharge decrease.

**Fig. 5:**
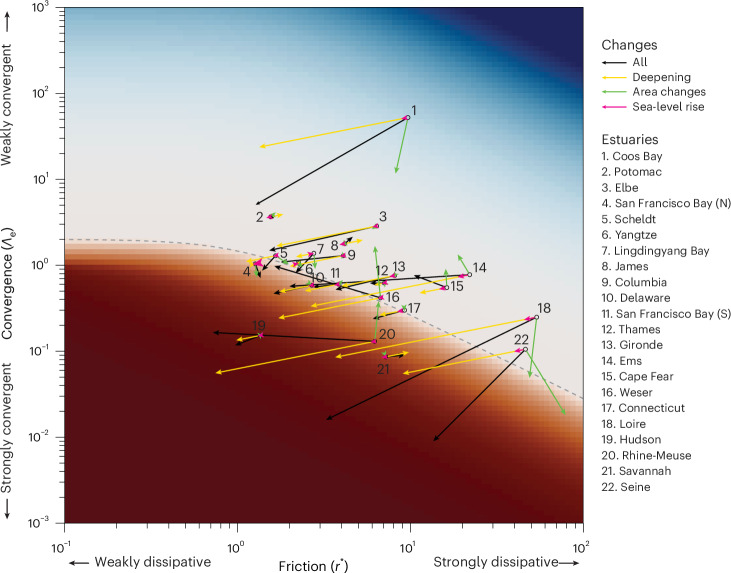
Estimated estuarine regime shifts based on a simplified analytical model. Background shading shows the amplification factor (*κ*_*i*_), with the grey dashed line marking the transition from tidal damping to tidal amplification. Arrows indicate the estimated shift in regime for 22 estuaries based on a simplified analytical model^74^, from the earliest available data (hollow circle at the base) to present-day conditions (arrowhead). Contributions from channel deepening, intertidal area loss and sea-level rise are separated along each arrow. Further details on the analytical model and assumptions are provided in Methods. The sensitivity of the amplification factor to changes in different parameters is highlighted in Extended Data Fig. 4. Model parameters are listed in Extended Data Tables 3 and 4.

**Extended Data Fig. 4:**
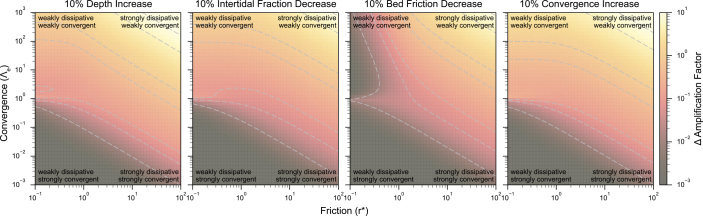
Sensitivity of tidal damping to estuarine parameter changes. Each panel shows the change in the tidal damping factor (*Δκ*_*i*_) across the dimensionless friction-convergence parameter space, resulting from a 10% change in a key estuarine property. From left to right: depth increase, intertidal fraction decrease, bed friction increase, and convergence increase (that is, decrease in convergence length). Contours indicate regions of stronger or weaker sensitivity. The amplification factor (*Δκ*_*i*_) and parameter space are derived from the analytical framework of^74^.

Although human interventions often exert a stronger influence on estuarine tides than river discharge, disentangling their individual effects and comparing them with sea-level rise remains difficult using field data alone. Interventions trigger a direct hydrodynamic response, followed by slower indirect effects such as morphological adaptation^65^. Because these processes unfold over long timescales, overlapping interventions complicate the isolation of individual impacts. We employ a simplified analytical model based on the framework of refs. ^64,74^ to establish the importance of channel deepening and area changes relative to the effect of sea-level rise in the estuaries we have studied. The model results demonstrate that most estuaries have shifted towards the weakly dissipative–strongly convergent regime (Fig. 5). Channel deepening emerges as the dominant driver, whereas area changes also plays an important role. Clearly, the effects of both types of human modification to estuaries overwhelm the effect of sea-level rise. Extended Data Fig. 4 shows that strongly dissipative, weakly convergent systems are most sensitive to change. Model parameters are listed in Extended Data Tables 3 and 4.

## Tidal change in the next century

**Fig. 6:**
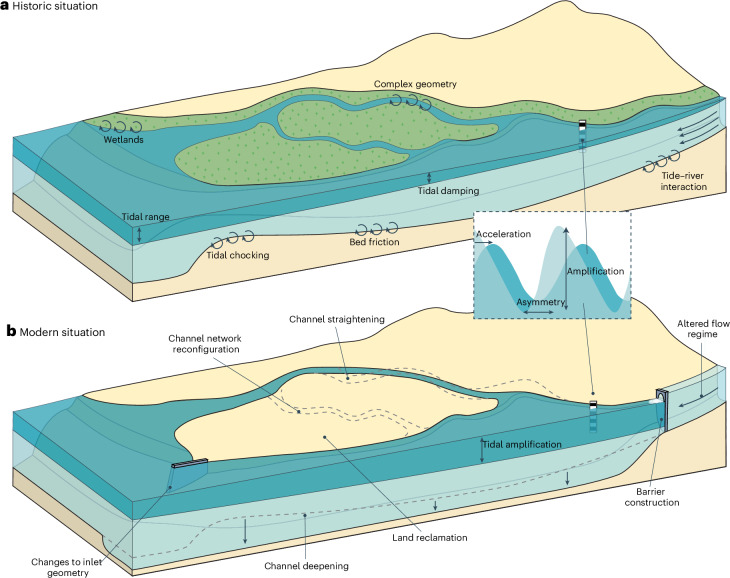
Conceptual diagram illustrating changes in tidal dynamics due to human interventions in an estuary. **a**, The historical state, where the tide dampens upstream due to various frictional losses, including bed friction, intertidal and wetland friction, turbulent flow through a complex geometry, tidal choking at the estuary mouth and interaction with upstream river discharge. **b**, The modern state, where human interventions, such as modifications to inlet geometry, channel network reconfiguration, channel straightening and deepening, land reclamation, barrier construction and an altered flow regime, have substantially reduced frictional losses. As a result, tides now typically amplify as they propagate upstream. The inset panel shows a time series of water levels at a tide gauge, highlighting that tides in the modern state have amplified, accelerated and become more asymmetric compared to the historical situation.

In two centuries, human interventions have tipped the balance between frictional damping and cross-section area convergence in favour of the latter (Fig. 6). Amplification of the tidal range, accelerated wave propagation and shifts in tidal asymmetry have altered the way tides propagate inland and interact with river and storm-driven processes. Many estuaries are still adjusting to past changes^65^. The slow pace of morphodynamic adaptation means that in systems where tidal amplification has been observed, further changes may still be unfolding. Past interventions have triggered feedback loops between estuarine hydrodynamics, morphology, ecology and continued human modifications^4,75^. As a result, even in the absence of additional human interventions, further tidal amplification, increased wave speeds and shifting asymmetry can be expected in many estuaries, especially due to the compounding effects of future sea-level rise^7,8^. This lagged response complicates management strategies, as past interventions continue to shape the current and future tidal regime^76^.

The human footprint on tides in estuaries impacts flood hazards by altering extreme water levels^77^, which are further modified by sea-level rise and climate change impacts on river discharge regimes and storm surges. Reduction in frictional losses improves river discharge conveyance, reducing extreme water levels during high discharge events^21^. Reduced friction amplifies tidal and storm surge propagation, increasing tide- and storm-driven flood risks. Therefore, a trade-off exists between upstream flood protection from fluvial flooding and increased vulnerability to coastal flooding^36^. Storm surge waves are expected to respond similarly to tidal waves in estuaries where deepening and land reclamation have diminished wave damping capacity.

Although sea levels at the estuary mouth have risen gradually, tidal amplification and local hydrodynamic modifications have driven much greater changes in water levels within the estuary. Subsidence aggravates tidal amplification and sea-level rise, and relative sea-level rise is expected to become an increasingly dominant driver of change. High-tide flooding, already a growing concern in many low-lying coastal cities, will become more frequent, as rising sea levels and tidal amplification compound^78^. This study underscores that sea-level rise rates measured at the estuary mouth poorly represent the mean water level changes further inland. Without intervention or morphological adaptation, rising sea levels will extend the influence of tides further upstream, potentially exacerbating salt intrusion into freshwater regions.

Restoring estuaries to a more natural state offers a promising strategy to reverse the trends observed in this study and enhance resilience to sea-level rise, storm surges, and high-tide flooding^79^. Nature-based approaches such as wetland restoration, managed realignment and reintroduction of intertidal storage can mitigate tidal amplification while providing co-benefits including carbon sequestration, improved water quality and biodiversity enhancement^80^. Unlike traditional hard engineering solutions that often exacerbate tidal amplification by reducing frictional damping, these adaptive strategies respond dynamically to rising sea levels and promote long-term sustainability. Changes in estuarine tides also alter tidal flow strength and asymmetry, key drivers of sediment transport and morphological evolution^30,31,81^. The loss of intertidal zones and wetlands reduces natural sediment deposition areas, while maintenance dredging increasingly dominates sediment dynamics, increasing turbidity and degrading water quality and ecosystems^82,83^. A shift in estuarine management is needed; away from interventions that amplify tidal energy and towards strategies that restore natural function, dampen tidal energy and safeguard ecosystem services under changing sea levels.

## Methods

### Estuarine characterization

We characterize estuarine tides through the along-channel profile of tidal range, wave propagation speed and the upstream river discharge. Morphodynamic characteristics include channel depth and planform area. To capture long-term changes, we aim to extend the temporal coverage of each estuary as far back as data availability permits. As a result, the time span analysed may differ among estuaries. European estuaries generally experienced high-impact human modifications earlier than Asian systems. In some estuaries, human interventions began before systematic measurements were established, but the most substantial changes probably occurred during and after industrialization, when the capacity to reshape estuarine systems increased markedly. Data originate from diverse sources and formats, requiring harmonization into a consistent dataset. Below, we outline the data compilation process and subsequent analysis methodology.

### Data collection and processing

Data were compiled from agency databases, scientific literature, maps and reports, encompassing tidal range, wave celerity, tidal asymmetry, channel depth, planform areas and discharge. As part of this process, we digitized several water level datasets and bathymetric charts (Fig. 1). Estuaries were selected primarily based on data availability, requiring at least four tide gauges along the estuary, including one near the mouth and sufficient spatial coverage to assess along-channel changes. As a result, the sample is biased towards larger, morphodynamically active systems with strong tidal and fluvial interactions, typically associated with major ports and long-term observational records. Lagoonal systems, fjords and wave-dominated estuaries are excluded due to their distinct dynamics. Smaller estuaries, lagoons^87^ and systems in underrepresented regions such as South America and Africa are not included, despite evidence that similar intervention histories and tidal changes may have occurred elsewhere^88^.

Given the heterogeneous nature of historical and modern datasets, we acknowledge several sources of uncertainty in our analysis. These include differences in vertical datum conventions, temporal coverage, spatial resolution and methodological approaches between estuaries. Many of these uncertainties are inherently difficult to quantify and extend beyond what can be captured through formal statistical estimates. For example, uncertainties in historical channel depth are not limited to spatial averaging but also arise from mapping choices, point density and interpolation techniques. Although some of these factors could be formally assessed, others lack sufficient metadata or documentation. Hydrodynamic variables such as the tidal range are generally robust across data sources, methodologies and averaging periods, with associated uncertainties typically on the order of a few centimetres, well below the magnitude of observed changes, which often span several decimetres to metres (for example, ref. ^89^). In summary, hydrodynamic variables are characterized by very limited uncertainty, whereas geometric variables may have larger associated uncertainties that are not easily captured by formal attribution methods. However, these uncertainties are not expected to affect the general trends observed in this study.

The following subsections describe standardization procedures applied to each variable.

#### Tidal range

Along-channel tidal range profiles were constructed using high-resolution and high–low water level records, annual mean data, published tidal datums and digitized maps. The tidal range was calculated as the difference between mean high and low water levels. Datums extracted from time series using the MATLAB Tide Peaks Toolbox^90^ or published figures were digitized using PlotDigitizer (https://plotdigitizer.sourceforge.net/). Whenever possible, multi-year averages were used to minimize the effects of nodal cycle variations and discharge influences. The streamwise distance of each station was determined by the curvilinear transformation of the centreline coordinates^91^.

Changes in tidal range were analysed by interpolating along-estuary tidal range profiles and identifying key parameters: tidal range at the estuary mouth, maximum tidal range and maximum change location.

#### High and low water levels

Mean high water (MHW) and mean low water (MLW) were calculated where sufficient data were available. Due to datum inconsistencies in historical records, MHW and MLW analyses were limited to selected upstream locations with consistent vertical references.

#### Wave celerity and tidal asymmetry

Wave celerity (*c*) was estimated as the ratio of the distance between the estuary mouth and the upstream tide gauge to the mean travel time of high and low waters:

1 $$c=\frac{{s}_{\mathrm{upstream}}}{0.5({t}_{\mathrm{HW}}+{t}_{\mathrm{LW}})},$$

where *s*_upstream_ is the streamwise coordinate of the upstream station and *t*_HW_ and *t*_LW_ are the travel times of high and low water, respectively. When only high-water travel times were available, high-water celerity was reported.

Tidal asymmetry was characterized by the ratio of high-water to low-water travel times:

2 $$\gamma =\frac{{t}_{\mathrm{HW}}}{{t}_{\mathrm{LW}}}=\frac{{c}_{\mathrm{LW}}}{{c}_{\mathrm{HW}}},$$

where *γ* represents the asymmetry parameter. Values less than 1 indicate flood dominance, whereas values greater than one indicate ebb dominance. This approach captures the asymmetry in tidal wave propagation over the full estuarine domain and is therefore well suited to assess system-scale changes. Where direct travel time data were unavailable, they were inferred from high- and low-water intervals in published tide tables.

For completeness, the tidal duration asymmetry, defined as the ratio of the duration of the falling tide to that of the rising tide, was also computed:

3 $${\gamma }_{\mathrm{dur}}=\frac{{t}_{\mathrm{fall}}}{{t}_{\mathrm{rise}}}.$$

This metric is shown in Extended Data Fig. 1 but is not discussed further in the main text.

#### Channel depth

Mean channel depth is calculated as the average depth of areas below the mean low water, referenced to the mean tide level. We compute this value for 2-km-long segments along the estuary’s streamline and subsequently average these values across the entire estuary. The depths are derived from bathymetric models, nautical charts or interpolated datasets. When referenced to different vertical datums, depths were adjusted using a mean tide level raster constructed from streamwise interpolation. Depth soundings and contour lines on nautical charts were interpolated to a digital elevation model (DEM) using the ArcGIS Topo to Raster tool.

#### Sea-level rise

To quantify relative sea-level rise near the mouths of each estuary, we used spatially distributed global sea-level reconstructions from ref. ^63^, which provide monthly water level estimates from 1880 to 2020. For each estuary, we identified the nearest available grid point to the mouth and extracted the corresponding time series of sea-level data. To estimate both the total amount and rate of sea-level rise over the relevant period, we applied a quadratic regression to the time series. This approach accounts for potential acceleration in sea-level rise and provides a smoothed estimate of long-term trends. Where possible, we selected a time window consistent with the availability of hydrodynamic and morphological data for each estuary. In cases where such data extended further back than 1880, we limited the analysis to 1880 onwards to match the temporal coverage of the sea-level dataset, assuming that the majority of sea-level rise occurred after this date. These estimates reflect regional sea-level changes and do not include local vertical land motion (for example, subsidence), which may lead to underestimation of relative sea-level rise in some estuaries. A summary of the derived rates and total rise values is provided in Supplementary Table 1.

#### Planform area

Subtidal, intertidal and supratidal zones were delineated using georeferenced nautical charts, bathymetric models or extracted from figures. The areas were computed using the Airy 1830 ellipsoid (EPSG:7001). The total area comparison excluded regions not consistently mapped across time periods. The precision of the supratidal extent varied depending on the delineation of the embankments (Supplementary Fig. 1).

#### Discharge

Mean annual discharge was estimated based on data from governmental agencies, scientific literature and technical reports. Where possible, discharge values were averaged over multiple years (up to a maximum of 30) to reduce the influence of interannual variability.

### Overview human interventions

On the basis of the data compiled in this study, previous literature and publicly available online sources, we identify seven key anthropogenic interventions that have influenced estuarine tidal dynamics in the systems analysed: channel deepening, land reclamation, tidal barrier construction, channel straightening, flow regime changes, inlet modifications and channel network reconfiguration. For each estuary, we tracked whether these interventions occurred during the period considered. An overview of the most notable interventions per system is provided in Supplementary Table (humaninterventions.xlsx), highlighting that estuarine evolution is often shaped by multiple and overlapping human modifications.

### Analytical model framework

To assess the tidal response of estuaries to anthropogenic interventions, we applied a simplified analytical model based on the framework developed by ref. ^74^. The model solves the linearized shallow-water equations for tidal propagation in exponentially converging estuaries, incorporating the effects of channel geometry and hydraulic drag. It yields a dimensionless dispersion relation that links estuarine convergence, friction and tidal amplification. The model is governed by two key dimensionless parameters:

- Estuarine convergence number (*Λ*_e_), which encapsulates the geometric configuration of the estuary, including convergence length, mean depth and intertidal area.
- Dimensionless hydraulic drag (r^*^), representing effective bottom friction, which may be reduced by deepening, bedform smoothing or suspended sediment-induced stratification.
- Dimensionless wave number (*κ*), whose imaginary component (*κ*_*i*_) determines the amplification or damping of the tidal wave.

For each estuary, we estimate *Λ*_e_ and r^*^ using the historical and modern bathymetric data, including channel depth, convergence length and intertidal area extent. These values were then used to calculate the dimensionless wave number (*κ*), whose imaginary component (*κ*_*i*_) determines the amplification or damping of the tidal wave. This allows us to assess the regime changes over time. The model assumes harmonic tidal forcing and neglects river discharge, salinity effects and nonlinearities, making it most suitable for comparing relative changes across systems rather than predicting absolute tidal behaviour.

This approach is conceptually similar to other analytical models of tidal propagation (for example, refs. ^15,92,93^). However, the model has limitations: it does not account for tidal reflections, nonlinear interactions or spatial variability in bathymetry and friction. As such, it cannot resolve tidal asymmetry or sediment transport dynamics in detail, nor can it simulate estuaries with a complex channel network, strong longitudinal inhomogeneity or complex boundary conditions. Despite these simplifications, the model provides a useful first-order estimate of tidal regime changes and the relative influence of deepening, area changes and sea-level rise. To estimate the relative influence of deepening, area changes and sea-level rise, we begin with the historic parameter values and adjust one component at a time. Deepening is represented by the total increase in channel depth minus the contribution from sea-level rise. Area changes are captured through modifications to estuarine convergence and intertidal storage fraction. Sea-level rise is incorporated by adjusting the historic channel depth, using estimated sea-level rise rates based on ref. ^63^ (Extended Data Table 1). This approach allows us to isolate the effect of each driver by modifying the relevant parameter while keeping all others at their historic values. A brief derivation of the analytical model is provided in Supplementary Methods. The full model equations and parameter definitions are available in ref. ^74^, and the specific parameter values used for each estuary are listed in Extended Data Tables 3 and 4.

## Online content

Any methods, additional references, Nature Portfolio reporting summaries, source data, extended data, supplementary information, acknowledgements, peer review information; details of author contributions and competing interests; and statements of data and code availability are available at 10.1038/s41561-026-01969-4.

## Supplementary information

Supplementary Information Supplementary Methods, Tables 1 and 2, and Fig. 1.

Supplementary Table (Sheet 1) This file lists all data sources used throughout the study. It includes information on the origin, type and additional details for each dataset referenced in the analysis. (Sheet 2) This file contains a detailed inventory of major human interventions that may have influenced the hydrodynamics of the estuaries analysed in this study. Each intervention is categorized by type, and relevant sources are provided.

## Figures and Tables

**Extended Data Table 1:**
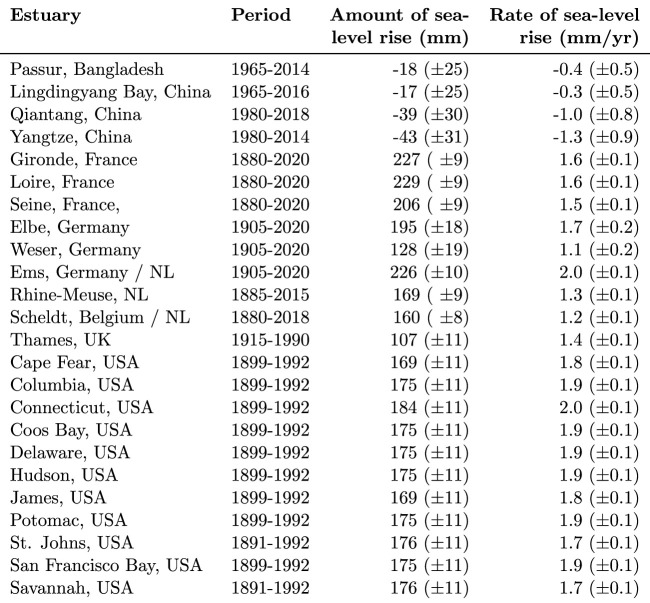
Overview of amounts and rates of sea-level rise

Estimates of sea-level rise near the mouths of the studied estuaries, based on^63^.Values are derived from a quadratic fit of the time series at the datapoint closest to each estuary mouth, with 95% confidence intervals shown in brackets.

**Extended Data Table 2:**
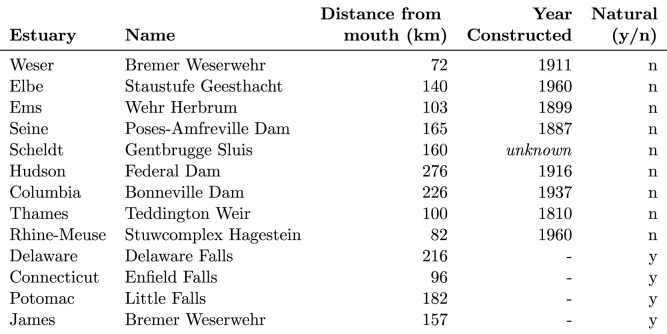
Overview of estuaries with tidal barriers

The table lists the name, approximate distance from the estuary mouth, year of construction (where applicable), and whether the barrier is natural or anthropogenic. Tidal barriers, defined as inland structures or natural features that restrict tidal propagation, are present in 13 of the 25 estuaries studied. Anthropogenic barriers include weirs, dams, and surge barriers, while natural barriers consist of geological features such as falls.

**Extended Data Table 3:**
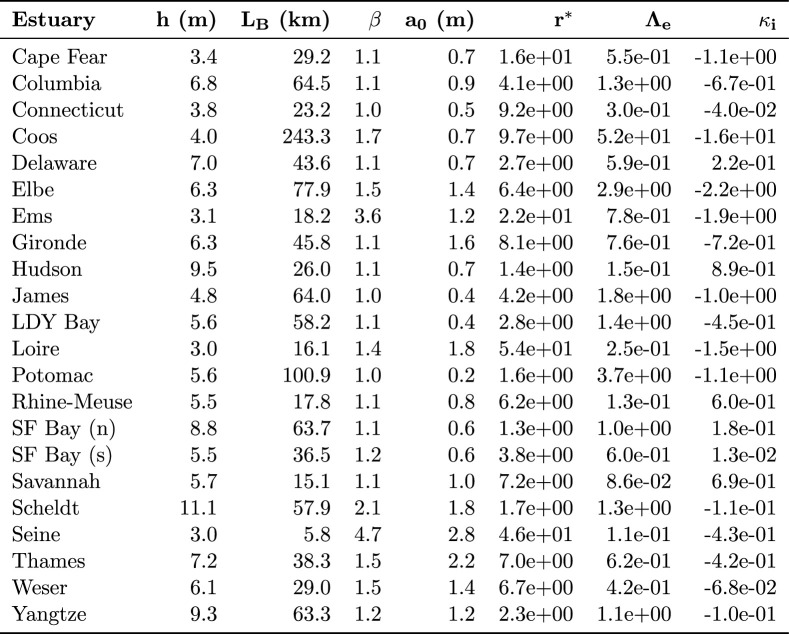
Model parameters for historic situation of the selected estuaries used in the analytical model of tidal wave propagation based on^74^

Parameters include channel depth (*h*), convergence length (L_b_), intertidal fraction (*β*), tidal amplitude at the mouth (a_0_), friction number (r^*^), convergence number (*λ*_e_), and amplification number (*κ*_i_). The amplification number *κ*_*i*_ is the imaginary part of the complex wave number and directly relates to tidal amplification (*κ*_*i*_ > 0) or damping (*κ*_*i*_ < 0). All values are computed assuming a uniform Chézy coefficient of 20m^1/2^/s.

**Extended Data Table 4:**
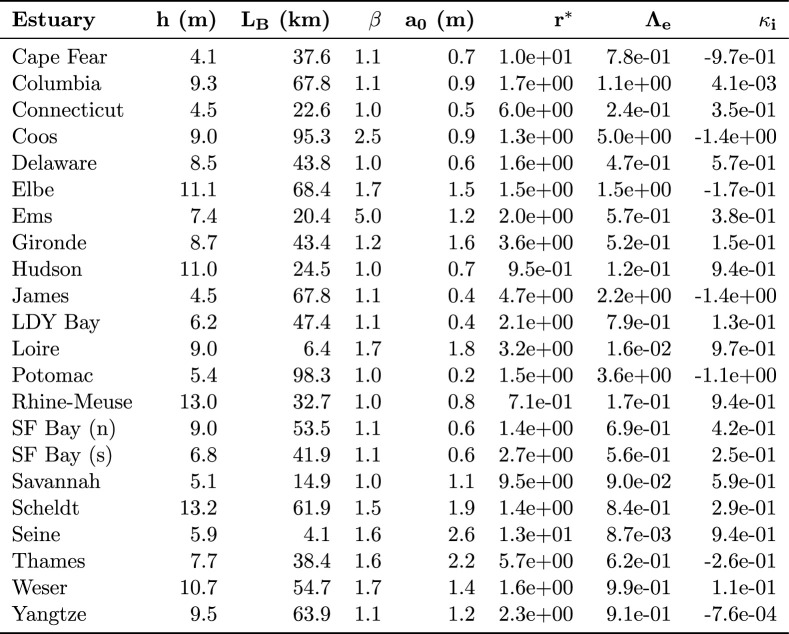
Model parameters for modern situation of the selected estuaries used in the analytical model of tidal wave propagation based on^74^

Parameters include channel depth (*h*), convergence length (*L*_*b*_), intertidal fraction (*β*), tidal amplitude at the mouth (*a*_0_), friction number (r^*^), convergence number (*λ*_*e*_), and amplification number (*κ*_*i*_). The amplification number *κ*_*i*_ is the imaginary part of the complex wave number and directly relates to tidal amplification (*κ*_*i*_ > 0) or damping (*κ*_*i*_ < 0). All values are computed assuming a uniform Chézy coefficient of 20m^1/2^/s.

## Data Availability

This study utilized a combination of previously published datasets and newly digitized data. A comprehensive list of all data sources is detailed in Supplementary Information (sources.xlsx). The availability and access conditions for these datasets are summarized as follows. Previously published data: due to licensing and copyright restrictions, the original datasets obtained from previous studies cannot be shared directly. Researchers interested in these datasets should refer to the sources listed in Supplementary Information (sources.xlsx) and contact the respective data holders for access. Digitized and derived data: the data digitized and derived datasets generated from analyses in this study are publicly available at 10.4121/54bf59a6-a827-4d50-a7ad-9f990b5aff89. For further enquiries or assistance regarding data access, please contact the corresponding author.

## Code availability

Custom scripts used for data processing, statistical analysis and figure generation were developed for this study. The analyses rely on standard statistical and signal-processing techniques described in the Methods section. The code is not organized as a general-purpose software package but is available from the corresponding author upon reasonable request.
