# Supplementary material for: Human footprint on estuarine tidal hydrodynamics
Source: Nat Geosci. 2026 Apr 24;19(6):620–9. doi: 10.1038/s41561-026-01969-4 (PMC13259921; doi:10.1038/s41561-026-01969-4)
Supplement: Supplementary file 1 — Supplementary Methods, Tables 1 and 2, and Fig. 1. [file 41561_2026_1969_MOESM1_ESM.pdf]

nature geoscience

Supplementary information https://doi.org/10.1038/s41561-026-01969-4

Human footprint on estuarine tidal hydrodynamics

In the format provided by the authors and unedited

Contents

- Supplementary Methods
- Supplementary Tables
- Supplementary Files
- Supplementary References

Supplementary Methods

Analytical Model Derivation and Amplification/Damping Factor

The analytical framework used in this study follows the linearized shallow-water formulation of [1, 2], describing tidal propagation in a narrow, exponentially converging estuary with intertidal area. The governing equations for mass and momentum conservation read:

$$(b_c + \Delta b)\frac{\partial \eta}{\partial t} + \frac{\partial (A_c u)}{\partial x} = 0, \quad \frac{\partial u}{\partial t} + g\frac{\partial \eta}{\partial x} + \frac{ru}{h} = 0, \tag{1}$$

where η is the tidal elevation, u the depth-mean velocity, b_c the channel width, Δb the width of the intertidal area, h the mean depth, $A_c \approx hb_c$ the flow area, and r a linearized friction coefficient related to the effective hydraulic drag C through $r = gU/C^2$ with a characteristic velocity amplitude U.
Assuming an exponentially converging width,

$$b_c = b_0\, e^{-x/L_b}, \tag{2}$$

with convergence length L_b, and harmonic oscillations of frequency ω,

$$\eta(x,t) = a_0 e^{i(\omega t - kx)}, \quad u(x,t) = U_0 e^{i(\omega t - kx - \phi)}, \tag{3}$$

substitution yields the dispersion relation

$$L_b k^2 - ik - \frac{(b_c + \Delta b)\omega^2}{ghb_c} L_b \left(1 - i\frac{r}{\omega h}\right) = 0. \tag{4}$$

Introducing the dimensionless quantities

$$\kappa = 2kL_b, \quad r^* = \frac{r}{\omega h}, \quad \Lambda_e = \frac{b_c + \Delta b}{b_c}\frac{4L_b^2\omega^2}{gh}, \tag{5}$$

A non-dimensional dispersion equation is obtained

$$\kappa^2 - 2i\kappa - \Lambda_e(1 - ir^*) = 0, \tag{6}$$

which provides the real and imaginary components of the complex wave number ($k = k_r + ik_i$):

$$\kappa_r = -\frac{1}{2}\left[2\sqrt{(\Lambda_e - 1)^2 + (\Lambda_e r^*)^2} + 2(\Lambda_e - 1)\right]^{1/2}, \tag{7}$$

$$\kappa_i = 1 \mp \frac{1}{2}\left[2\sqrt{(\Lambda_e - 1)^2 + (\Lambda_e r^*)^2} - 2(\Lambda_e - 1)\right]^{1/2}. \tag{8}$$

Here, Λ_e is the *estuarine convergence number*, summarizing the effects of convergence, depth, and intertidal storage, while r^* represents the *dimensionless effective hydraulic drag.* The imaginary part of the complex wave number κ_i determines the local amplification or damping of the tidal wave along the estuary. $\kappa_i > 0$ corresponds to amplification and $\kappa_i < 0$ to damping of the tidal amplitude.

Supplementary Tables

Key hydrodynamic and geometric data

Table S1 summarizes the key hydrodynamic and geometric characteristics of the estuaries analyzed in this study,

Table S1 | Overview of estuaries with key hydrodynamic and geometric parameters. Values are based on historical and modern datasets; see Methods and Appendix D for data sources, standardization procedures, and time periods represented.

Estuary	Period	Tidal range at mouth (m)	Max tidal range (m)	Max change tidal range (m)	Location max tidal range (km)	Location max change tidal range (km)	Wave propagation speed (m/s)	Asymmetry Parameter (-)	Channel Depth (m -MTL)	Total area (km²)	Subtidal area (km²)	Intertidal area (km²)	Supratidal area (km²)	Annual mean discharge (m³/s) - 95% CI indicated where possible
**Passur, Bangladesh**	1960-1965	2.71	2.89	1.54	102	147	7.0	0.78	Unknown	1497*	332*	0*	1165*	Unknown
	2011-2014	2.72	3.63	3.01	102	147	11.4	0.83	9.73	174*	174*	0*	0*	1275
		0.01 (0%)	0.74 (26%)	1.47 (95%)	0		4.4 (63%)	0.05		-1323 (88%)	-158 (48%)	0 (0%)	-1165 (-100%)	
**Lingdingyang Bay, China**	1955-1970	0.80	1.40	1.40	95	95	5.3	0.95	5.1	2198	1901	297	Unknown	Unknown
	2012-2019	0.78	1.52	1.52	95	95	6.3	0.80	5.3	1837	1701	136	Unknown	Unknown
		-0.02 (-3%)	0.12 (9%)	0.12 (9%)	0		1.0 (19%)	-0.15	0.2 (3%)	-361 (-16%)	-200 (-11%)	-161 (-54%)		
**Qiantang, China**	1976-1984	1.92	5.71	2.40	131	179	Unknown	Unknown	Unknown	7843	Unknown	Unknown	Unknown	Unknown
	2010-2018	2.13	6.41	3.16	131	179	Unknown	Unknown	Unknown	7067	Unknown	Unknown	Unknown	Unknown
		0.21 (10%)	0.70 (11%)	0.76 (32%)	0					-776 (-10%)				
**Yangtze, China**	1958-1987	2.37	2.37	1.65	0	160	9.2	0.95	7.83	4198	3747	451	Unknown	29200 (±1900)
	2002-2014	2.37	2.37	1.80	0	160	10.0	0.95	7.85	3668	3416	252	Unknown	26900 (±2000)
		0 (0%)	0.00 (0%)	0.15 (9%)	0		0.9 (9%)	0	0.02 (0%)	-530 (-13%)	-331 (-9%)	-199 (-44%)		-2300 (-8%)
**Gironde, France**	1825	3.17	4.03	4.03	52	94	6.4	0.66	6.5	527	462	65	68	Unknown
	1994-2024	3.19	4.52	4.52	94	94	9.7	0.67	8.5	522	436	86	23	816 (±99)
		0.02 (1%)	0.49 (12%)	0.49 (12%)	42		3.3 (51%)	0.00	2.1 (32%)	-5 (-1%)	-26 (-6%)	20 (31%)	-46 (-67%)	
**Loire, France**	1821-1892	3.53	3.53	1.26	0	48	3.5	0.53	3.2	260	131	34	95	783 (±80)
	1994-2024	3.68	4	3.87	25	48	11.2	0.41	8.6	159	79	43	36	785 (±87)
		0.15 (4%)	0.47 (13%)	2.61 (207%)	25		7.6 (216%)	-0.11	5.4 (168%)	-101 (-39%)	-52 (-40%)	9 (28%)	-59 (-62%)	2 (0%)
**Seine, France**	1834	5.52	5.56	0.77	13	63	5.4	0.61	3.0	386	109	277	22	Unknown
	1994-2023	5.28	5.28	3.24	1	63	6.2	0.98	5.9	128	77	52	Unknown	480 (±47)
		-0.24 (4%)	-0.28 (-5%)	2.47 (321.09%)	-12		0.9 (17%)	0.37	3.0 (101%)	-258 (-67%)	-32 (-30%)	-225 (-81%)		
**Elbe, Germany**	1898-1910	2.85	2.85	0.85	0.0	116	5.5	0.74	7.0	642	297	184	162	676 (±22)
	1987-2023	2.93	3.83	3.60	108	116	7.9	0.85	9.3	495	227	205	63	689 (±35)
		0.08 (3%)	0.98 (34%)	2.75 (324%)	108		2.4 (44%)	0.11	2.4 (44%)	-147 (-23%)	-70 (-23%)	22 (12%)	-99 (-61%)	12 (2%)
**Weser, Germany**	1898-1910	2.72	3.32	1.39	48	115	9.7**	Unknown	6.6	252	84	111	56	325 (±12)
	1987-2023	2.87	4.27	4.27	115	115	17.5**	0.75	9.5	203	58	120	25	323 (±16)
		0.15 (6%)	0.95 (29%)	2.88 (207%)	68		7.8 (80%)		2.9 (-20%)	-49 (-31%)	-26 (-31%)	9 (8%)	-32 (-57%)	-2 (-0%)
**Ems, The Netherlands / Germany**	1898-1937	2.49	3.02	0.55	44	97	5.8	0.74	5.1	380	169	178	34	Unknown
	1987-2023	2.40	3.70	3.19	84	97	9.2	0.64	7.4	323	105	191	28	82 (±4)
		-0.09 (-4%)	0.68 (23%)	2.64 (480%)	40		3.3 (57%)	-0.10	2.3 (46%)	-57 (-15%)	-64 (-38%)	13 (7%)	-6 (-18%)	
**Rhine-Meuse, The Netherlands**	1879-1890	1.67	1.67	1.30	0	19	3.1	0.71	5.4	91	60	7	24	833
	2006-2022	1.58	1.58	1.50	0	19	5.4	0.55	13.2	70	70	0	0	1480
		-0.09 (-5%)	-0.09 (-5%)	0.20 (17%)	0		2.3 (74%)	-0.15	7.8 (144%)	-21 (-23%)	10 (17%)	-7 (-96%)	-24 (-100%)	647 (78%)
**Scheldt, The Netherlands / Belgium**	1799-1890	3.62	4.19	3.40	78	107	6.7**	Unknown	11.1	503	248	216	40	Unknown
	2013-2022	3.76	5.20	4.83	89	107	10.4**	Unknown	13.2	324	210	80	34	135 (±13)
		0.14 (4%)	1.01 (24%)	1.43 (42%)	11		3.7 (55%)		2.1 (±0.6;19%)	-179 (-36%)	-38 (-15)	-136 (-63%)	-5 (-13%)	
**Thames, United Kingdom**	1900-1929	4.37	5.57	5.34	68	55	7.1	0.52	7.5	108	67	41	Unknown	74 (±9)
	1975-2004	4.41	5.76	5.55	69	55	6.0	0.38	7.8	109	63	46	Unknown	62 (±8)
		0.04 (1%)	0.19 (3%)	0.21 (4%)	1		-1.1 (-15%)	-0.14	0.3 (4%)	1 (1%)	-4 (-6%)	5 (12%)		2 (3%)
**Cape Fear, USA**	1855-1939	1.31	1.37	0.73	4	44	3.4	0.87	3.4	137	105	10	22	Unknown
	1934-2024	1.37	1.37	1.30	0	44	4.2	0.99	4.2	131	86	15	30	184 (±30)
		0.06 (5%)	0.00 (0%)	0.57 (78%)	-4		0.80 (24%)	0.12	0.8 (23%)	-6 (-4%)	-19 (-18%)	6 (58%)	8 (35%)	
**Columbia, USA**	1874-1925	1.88	1.98	0.24	15	166	6.1	0.83	4.9	899	560	60	280	6110 (±193)
	1983-2023	1.78	2.07	0.74	25	166	7.1	0.73	6.3	615	486	79	50	5023 (±183)
		-0.10 (-5%)	0.09 (5%)	0.50 (208%)	10		1.0 (16%)	-0.10	1.5 (30%)	-285 (-32%)	-74 (-13%)	19 (32%)	-230 (-82%)	-1088 (-18%)
**Connecticut, USA**	1895-1934	0.96	0.96	0.25	0	73	4.1	0.73		74	52	4	18	385 (±9)
	1969-2024	0.97	1.01	0.59	1	73	4.1	0.68		71	53	3	15	450 (±17)
		0.01 (1%)	0.05 (5%)	0.34 (137%)	1		0.0 (0%)	-0.05		-2 (-3%)	0 (1%)	-1 (-14%)	-2 (-13%)	65 (17%)
**Coos Bay, USA**	1895-1899	1.48	1.48	1.27	0	54	9.9	0.86	4.0	55	25	19	12	Unknown
	1983-2024	1.76	2.05	1.97	80	54	16.8	1.27	8.6	43	18	21	4	Unknown
		0.28 (19%)	0.57 (39%)	0.70 (55%)	80		6.9 (70%)	0.41	4.6 (117%)	-13 (-23%)	-7 (-28%)	2 (10%)	-8 (65%)	
**Delaware, USA**	1847-1922	1.30	1.98	1.36	112	212	6.3	0.82	7.0	2601	1996	45	560	327 (±11)
	1983-2024	1.24	2.49	2.49	212	212	8.1	0.79	8.5	2534	2041	32	460	374 (±21)
		-0.06 (-5%)	0.51 (26%)	1.13 (83%)	100		1.7 (27%)	-0.03	1.5 (22%)	-68 (-3%)	46 (2%)	-13 (-29%)	-100 (-18%)	46 (14%)
**Hudson, USA**	1863-1955	1.40	1.60	0.70	13	264	6.7	0.86	9.4	1195	1003	21	170	206 (±7)
	1983-2024	1.43	1.52	1.52	264	264	7.8	0.90	10.9	1002	935	27	41	252 (±9)
		0.03 (2%)	-0.08 (-5%)	0.82 (117%)	251		1.1 (17%)	0.04	1.5 (16%)	-193 (-16%)	-69 (-7%)	6 (26%)	-130 (-76%)	45 (22%)

Table S1 Continued | Overview of estuaries with key hydrodynamic and geometric parameters. Values are based on historical and modern datasets; see Methods and Appendix D for data sources, standardization procedures, and time periods represented.

Estuary	Period	Tidal range at mouth (m)	Max tidal range (m)	Max change tidal range (m)	Location max tidal range (km)	Location max change tidal range (km)	Wave propagation speed (m/s)	Asymmetry Parameter (-)	Channel Depth (m -MTL)	Total area (km²)	Subtidal area (km²)	Intertidal area (km²)	Supratidal area (km²)	Annual mean discharge (m³/s)
**James, USA**	1882-1928	0.76	1.22	1.13	168	149	5.3	0.86	4.9	722	605	10	107	210 (±8)
	1919-2024	0.74	1.01	0.94	156	149	5.3	0.88	4.5	699	611	7	82	199 (±13)
		-0.02 (-2%)	-0.21 (-17%)	-0.19 (-17%)	-12		0.0 (0%)	0.02	-0.3 (-7%)	-23 (-3%)	6 (1%)	-4 (-35%)	-25 (-24%)	-11 (-5%)
**Potomac, USA**	1884-1905	0.43	0.94	0.61	179	58	6.8	0.96	5.6	1200	1164	4	32	269 (±13)
	1953-2024	0.38	0.85	0.50	170	58	6.9	0.91	5.5	1191	1162	9	19	285 (±23)
		-0.05 (-12%)	-0.09 (-10%)	-0.11 (-18%)	-9		0.1 (2%)	-0.05	-0.2 (-3%)	-10 (-1%)	-2 (-0%)	5 (136%)	-13 (-41%)	16 (6%)
**St. Johns, USA**	1884-1898	1.50	1.50	0.54	0	21	5.5	0.99	5.1	150	84	2	64	Unknown
	1983-2024	1.47	1.47	0.93	0	21	5.8	0.92	8.6	126	72	19	34	142 (±20)
		-0.03 (-2%)	-0.03 (-2%)	0.39 (72%)	0		0.3 (5%)	-0.07	3.5 (69%)	-24 (-16%)	-12 (-14%)	17 (714%)	-30 (-47%)	
**San Fransisco North Bay, USA**	1850-1899	1.13	1.50	1.50	59	59	5.2	0.78	9.2	1270	677	86	507	1079 (±109)
	1970-2011	1.25	1.39	1.13	27	59	5.4	0.82	9.3	1022	605	50	366	749 (±112)
		0.12 (11%)	-0.11 (-7%)	-0.37 (-24%)	-31		0.2 (4%)	0.04	0.1 (2%)	-249 (-20%)	-71 (-11%)	-36 (-42%)	-141 (-28%)	-331 (-31%)
**San Fransisco South Bay, USA**	1850-1899	1.13	1.86	1.84	45	55	13.7	0.70	5.6	813	433	118	262	Unknown
	1970-2001	1.25	2.32	2.10	69	55	12.8	0.63	7.0	557	434	68	55	Unknown
		0.12 (11%)	0.46 (25%)	0.26 (14%)	24		-0.9 (-7%)	-0.06	1.4 (25%)	-256 (-32%)	1 (0%)	-50 (-43%)	-207 (79%)	
**Savannah, USA**	1855-1939	2.06	2.06	1.17	0	33	3.4	0.75	4.4	141	61	7	72	306 (±15)
	1937-2023	2.11	2.48	2.44	30	33	5.4	0.72	5.2	128	61	8	59	288 (±18)
		0.05 (2%)	0.42 (20%)	1.27 (109%)	30		2.1 (61%)	-0.02	0.8 (17%)	-13 (-9%)	0 (0%)	1 (10%)	-13 (-18%)	-19 (-6%)

*** Values refer only to the poldered area north of the Sundarbans; including the Sundarbans would reduce the relative area change.**
**** Values refer to the propagation speed of the high-water peak.**
† In cases where long time intervals are shown, this typically indicates that parts of the most recent bathymetric data are already several years old. Areas subject to rapid change—such as navigational fairways—are generally surveyed more frequently.

Digitization

Table S2 provides an overview of the datasets digitized for this study. In addition to water level time series and digital elevation models, other variables, such as tidal datums, reclaimed areas, and travel times, were also digitized from tide tables, reports, scientific publications, and historical maps.

Table S2 | Overview of Digitized Historical Data Sources.

Location	Variables Digitzed	Year	Original Source	Digitization Details
Gironde, France	Water level time series, tidal range, propagation speeds	1825	*Pilote Francais Marées. Hautes Mers et Basses Mers Observées pendant les campagnes hydrographiques de 1816 à 1838*	High-low water level time series for 6 tide gauges along the Gironde were extracted from the original source.
Loire, France	Water level time series, tidal range, propagation speeds	1821	*Pilote Francais Marées. Hautes Mers et Basses Mers Observées pendant les campagnes hydrographiques de 1816 à 1838*	High-low water level time series for 6 tide gauges along the Loire were extracted from the original source.
Seine, France	Water level time series, tidal range, propagation speeds	1834	*Pilote Francais Marées. Hautes Mers et Basses Mers Observées pendant les campagnes hydrographiques de 1816 à 1838*	High-low water level time series for 7 tide gauges along the Seine were extracted from the original source.
	Bathymetry, planform areas, channel depth	1834	Beautemps-Beaupré, C.-F. (1841). *Carte particulière des Côtes de France. Cours de la Seine depuis le Trait jusqu'à Honfleur.* [Nautical Chart].<br><br>Beautemps-Beaupré, C.-F. (1841). *Carte Des Cotes de France. Partie Comprise Entre Dives et Saint Valery-en-Caux, Embouchure de la Seine.* [Nautical Chart].	Depth soundings, contours, and planform areas were digitized. Depth soundings and contours from these charts were digitized to a digital elevation model
Elbe, Germany	Bathymetry, planform areas, channel depth	1905	Deutsches Hydrographisches Institut. (1905). *Mündungen der Jade, Weser & Elbe* [Nautical Chart].<br><br>Deutsches Hydrographisches Institut. (1905). *Die Elbe von Brunsbüttel Bis Krautsand* [Nautical Chart]<br><br>Deutsches Hydrographisches Institut. (1905). *Die Elbe von Krautsand Bis Brunshausen* [Nautical Chart].<br><br>Deutsches Hydrographisches Institut. (1905). *Die Elbe von Brunshausen Bis Tinsdahl* [Nautical Chart].<br><br>Deutsches Hydrographisches Institut. (1905). *Die Elbe von Tinsdahl Bis Hamburg* [Nautical Chart].	Depth soundings, contours, and planform areas were digitized. Depth soundings and contours from these charts were digitized to a digital elevation model.
Weser, Germany	Bathymetry, planform areas, channel depth	1905	Deutsches Hydrographisches Institut. (1905). *Mündungen der Jade, Weser & Elbe* [Nautical Chart].<br><br>Deutsches Hydrographisches Institut. (1905). *Die Weser von Bremerhaven bis Elsfleth* [Nautical Chart].<br><br>Deutsches Hydrographisches Institut. (1905). *Die Weser von Elsfleth bis Bremen* [Nautical Chart].	Depth soundings, contours, and planform areas were digitized. Depth soundings and contours from these charts were digitized to a digital elevation model.
Rhine-Meuse, The Netherlands	Bathymetry, planform areas, channel depth	1884	Ministerie van Marine, afdeling Hydrographie (1884) *De Maas tot Rotterdam. Hydrografische kaart op de schaal van 1:30000* [Nautical Chart].	Depth soundings, contours, and planform areas were digitized. Depth soundings and contours from these charts were digitized to a digital elevation model.
Connecticut, USA	Bathymetry, planform areas, channel depth	1895-1911	U.S. Coast and Geodetic Survey. (1911). *253 Connecticut River: Entrance to Deep River* [Nautical Chart].<br><br>U.S. Coast and Geodetic Survey. (1895). *254 Connecticut River: Deep River to Higganum* [Nautical Chart].<br><br>U.S. Coast and Geodetic Survey. (1895). *255 Connecticut River: Higganum to Rocky Hill* [Nautical Chart].<br><br>U.S. Coast and Geodetic Survey. (1895). *256 Connecticut River: Rocky Hill to Hartford* [Nautical Chart].	Depth soundings, contours, and planform areas were digitized. Depth soundings and contours from these charts were digitized to a digital elevation model.
James, USA	Bathymetry, planform areas, channel depth	1882-1888	U.S. Coast and Geodetic Survey. (1884). *401a James River: Hampton Roads to Point of Shoals (sheet 1)* [Nautical Chart].<br><br>U.S. Coast and Geodetic Survey. (1882). *401b James River: Point of Shoals to Sandy Point (sheet 2)* [Nautical Chart].<br><br>U.S. Coast and Geodetic Survey. (1882). *401c James River: Sandy Point to City Point (sheet 3)* [Nautical Chart].<br><br>U.S. Coast and Geodetic Survey. (1888). *401d James River: City Point to Kingsland Creek (sheet 4)* [Nautical Chart].<br><br>U.S. Coast and Geodetic Survey. (1888). *401e James River: Kingsland Creek to Richmond (sheet 5)* [Nautical Chart].	Depth soundings, contours, and planform areas were digitized. Depth soundings and contours from these charts were digitized to a digital elevation model.
Potomac, USA	Bathymetry, planform areas, channel depth	1884	U.S. Coast and Geodetic Survey. (1884). *388 Potomac River: From Entrance to Piney Point (sheet 1)* [Nautical Chart].<br><br>U.S. Coast and Geodetic Survey. (1884). *389 Potomac River: Piney Point to Lower Cedar Point (sheet 2)* [Nautical Chart].<br><br>U.S. Coast and Geodetic Survey. (1884). *390 Potomac River: Lower Cedar Point to Indian Head (sheet 3)* [Nautical Chart].<br><br>U.S. Coast and Geodetic Survey. (1884). *391 Potomac River: Indian Head to Georgetown (sheet 4)* [Nautical Chart].	Depth soundings, contours, and planform areas were digitized. Depth soundings and contours from these charts were digitized to a digital elevation model.

Supplementary Figures

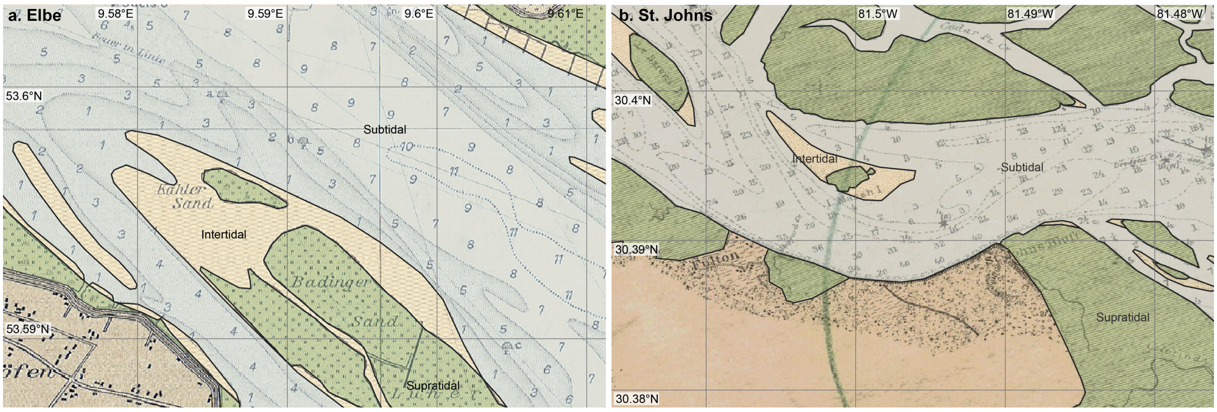

Figure 1: **Examples of areal extent determination from nautical charts.** (a) A section of the Elbe River with clearly indicated embankments (after Deutsches Hydrographisches Institut [3]); (b) a section of the St. Johns Estuary, where delineating the supratidal area is more challenging (after U.S. Coast and Geodetic Survey [4], from NOAA's Historical Map & Chart Collection).

Supplementary Files

humaninterventions.xlsx - This file contains a detailed inventory of major human interventions that may have influenced the hydrodynamics of the estuaries analyzed in this study. Each intervention is categorized by type, and relevant sources are provided.

sources.xlsx - This file lists all data sources used throughout the study. It includes information on the origin, type, and additional details for each dataset referenced in the analysis.

References (Supplementary Materials)

1. Winterwerp, J. C. & Wang, Z. B. Man-induced regime shifts in small estuaries—I: theory. *Ocean Dynamics* **63,** 1279–1292 (2013).
2. Winterwerp, J. C. & Wang, Z.-B. Hydrosedimentological response to estuarine deepening: Conceptual analysis. *Journal of Waterway, Port, Coastal, and Ocean Engineering* **147,** 04021023 (2021).
3. Deutsches Hydrographisches Institut. Die Elbe von Brunshausen bis Tinsdahl. Nautical chart. Hamburg, Germany, 1905.
4. U.S. Coast and Geodetic Survey. 454A St. Johns River: From Entrance to Jacksonville. Nautical chart. 1898.
